# Feasibility of Spinal Anesthesia Placement Using Automated Interpretation of Lumbar Ultrasound Images: A Prospective Randomized Controlled Trial

**DOI:** 10.4172/2155-6148.1000878

**Published:** 2019-02-25

**Authors:** Priyanka Singla, Adam J Dixon, Jessica L Sheeran, David Scalzo, Frank W Mauldin, Mohamed Tiouririne

**Affiliations:** 1 Department of Anesthesiology, University of Virginia Health System, Charlottesville, VA 22908, Virginia, USA; 2 Rivanna Medical, LLC, 107 E Water St, Charlottesville, VA 22902, Virginia, USA; 3 Department of Biomedical Engineering, University of Virginia, Charlottesville, VA 22908, Virginia, USA

**Keywords:** Spinal anesthesia, Neuraxial ultrasound, Bone-specific imaging

## Abstract

**Background:**

This study evaluated the efficacy of spinal anesthesia administration by resident physicians when using an ultrasound system with automated neuraxial landmark detection capabilities.

**Methods:**

150 patients were enrolled in this trial. Anesthesiology residents placed spinals in subjects undergoing scheduled cesarean delivery using one of three techniques to identify neuraxial landmarks: palpation, ultrasound, or combined palpation and ultrasound. Ultrasound was performed using a handheld system that automatically identified neuraxial landmarks (e.g. midline, intervertebral spaces). All residents watched a 10-minute video and received 20 minutes of hands-on training prior to participating in the study. First insertion success rate was the primary end point.

**Results:**

Among all patients, use of ultrasound resulted in a 11% greater first-insertion success rate (RR: 1.11 [0.85–1.47], p=0.431), a 15% reduction in needle insertions (RR: 0.85, p=0.052), and a 26% decrease in needle passes (RR: 0.74, p=0.070). In obese patients of BMI ≥ 30 kg/m^2^, use of ultrasound resulted in 26% greater first-insertion success rates (RR: 1.26, p=0.187), a 21% decrease in needle insertions (RR: 0.79, p=0.025), a 38% decrease in needle passes (RR: 0.62, p=0.030), and a 75% decrease in patients reporting neutral or low patient satisfaction with anesthesia administration (RR: 0.25, p=0.004).

**Discussion:**

Resident anesthesiologists competently utilized the ultrasound system after receiving minimal training. Technical endpoints and patient satisfaction trended towards improvement when ultrasound was used prior to spinal placement, with stronger trends observed in obese patients. Additional study is required to fully characterize the impact of the ultrasound system on clinical efficacy.

## Introduction

Spinal anesthesia is commonly administered using the landmarks technique, in which the skin is palpated to locate the midline and vertebral level [1]. Procedural success relies on correct identification of anatomic landmarks and practitioner experience [2]. However, identifying landmarks is often difficult due to patient conditions such as obesity, scoliosis, and edema [1,3], leading to multiple needle insertion attempts [1] and complications [4]. Radiographic imaging modalities can provide accurate anatomic information but are not safe to use during pregnancy.

Consequently, ultrasound has been advocated as an adjunct to obstetric neuraxial procedures [1,3,5,6]. Use of neuraxial ultrasound enables confirmation of the vertebral level [7–9], estimation of the depth to the epidural space [5,7,10–12] and provides an indication of the needle angulation required to advance the needle to the interlaminar space. Together, these benefits likely contribute to an improved safety profile [13], and multiple meta-analyses and studies have confirmed that use of neuraxial ultrasound results in fewer needle insertions, fewer exploratory needle passes, and shorter times required to complete the needle placement procedure, especially in obese patient [1,6,7,14,15].

Successful use of neuraxial ultrasound is heavily dependent on user skill and experience [16,17] and training regimens required to gain competency are demanding, often requiring prolonged periods of expert supervision and instruction [15–18]. Limitations in existing ultrasound imaging technologies contribute to this learning curve, as bones are imaged poorly and neuraxial images are difficult to interpret [19], particularly in obese patients [12]. Attempts to address these limitations have included techniques to enhance bone image quality [20] and the development of landmark recognition algorithms that automatically detect anatomical features within ultrasound images [21,22]. However, until recently [10,23], these approaches have not been validated clinically.

Many studies that have evaluated the use of neuraxial ultrasound in the obstetric setting have controlled for variability in provider expertise by having clinicians who are experienced in the technique perform the preprocedural ultrasound examination [1,5,14,24–26]. In many cases, the results of the ultrasound exam are relayed in the form of skin markings to a separate care provider who completes the neuraxial procedure. Unfortunately, studies of design are not reflective of workflows in routine clinical practice, and questions remain as to how generalizable the results are to settings in which experienced providers are not available to perform the ultrasound examination.

To address this limitation, the aim of the present study was to evaluate the feasibility of implementing neuraxial ultrasound as a means of identifying the needle insertion location prior to needle placement in a cohort of resident physicians with minimal ultrasonography training. The ultrasound system incorporates bone-specific imaging modes and also automatically identifies neuraxial landmarks, including the midline and intervertebral spaces [21]. The technology is intended to simplify image interpretation and reduce the learning curve required to implement neuraxial ultrasonography.

## Patients and Methods

This prospective RCT was approved by the University of Virginia IRB (HSR# 18070) and all subjects provided written informed consent. The trial was registered at clinicaltrials.gov (NCT02442973 and NCT03090295, release date May 13, 2015). This manuscript and study adhere to CONSORT guidelines.

Patients undergoing elective cesarean delivery at UVA Medical Center between January 2016 and October 2017 were eligible to participate. Inclusion criteria were: age between 18 and 45, ASA I–III, and scheduled cesarean delivery under spinal or combined spinal epidural anesthesia. Patients were excluded if they had self-reported or known spinal deformities, prior lumbar spine surgery, or allergy to ultrasound gel. Patients were randomized to one of three experimental groups: palpation-only group (P), ultrasound-only group (U), or palpation plus ultrasound group (PU). The randomization sequence was maintained by the research coordinator and was tabulated using a block randomization procedure prior to commencing the study. The group assignment for each patient was concealed from the clinical practitioners until 30 minutes prior to each procedure.

Needle puncture site identification was performed differently in each group. In Group P, the needle entry location was identified using palpation. The intercristal line was palpated to identify the L4-L5 intervertebral space and midline was determined by palpating spinous processes. In Group U, the needle entry point was located using Accuro^®^ SpineNav3D^™^ mode (Rivanna Medical, Charlottesville, VA). The sacrum was scanned in the transverse plane and the Accuro was translated in the cephalad direction to identify the L4-L5 interspace. Residents were instructed to use SpineNav3D to identify midline and to mark the needle insertion location via physical indentation after Accuro indicated the presence of the L4-L5 interspace. Indentation marks were made using the Accuro Locator™, a detachable skin marking tool that clipped onto Accuro. The depth estimate to the epidural space produced by SpineNav3D was recorded and an image of the L4-L5 interspace was saved. Residents were not permitted to palpate for landmarks in Group U. In Group PU, ultrasound scanning was performed as in Group U and was supplemented by palpation as performed in Group P. Accuro imaging technologies are described in Tiouririne et al. [21,22].

During spinal placement, patients were seated with shoulders slouched and head flexed to expose the lower back. After marking the needle insertion location, the skin was cleaned, disinfected, and infiltrated with 1% lidocaine. The needle was inserted and the spinal dose was administered after confirmation of correct placement by CSF aspiration. The depth of the spinal needle to the subarachnoid space was recorded by measuring the distance from the needle tip to where it exited the skin with a ruler. All procedures were performed by second through fourth year anesthesiology residents under the supervision of an anesthesiology attending. In all groups, the number of needle insertion attempts by a resident was limited to a maximum of three, after which point the attending took over. Each resident performed up to a maximum of 10 cases, split across all groups.

Prior to using Accuro, residents watched a 10-minute training video and scanned one volunteer under the supervision of an attending anesthesiologist to gain familiarity with the device. Training was not intended to develop competency in neuraxial ultrasonography techniques, but rather to explain the user interface of the Accuro. Training was repeated if more than 5 days passed between either the initial training or any subsequent use of Accuro. The primary outcome was the rate of successful dural puncture on the first insertion attempt. Complete withdrawal of the spinal or introducer needle from the patient’s skin constituted an additional insertion attempt. Needle passes were defined as any change in the needle trajectory that did not involve removal of the spinal needle from the skin.

Secondary outcomes included: number of insertions, number of passes, time taken to establish landmarks (i.e. time between start of landmark identification and needle insertion), needle insertion time (i.e. time between initial needle insertion and visualization of CSF), total procedure time (i.e. time between the start of landmark identification and ending when T4 sensory level was achieved), patient satisfaction with anesthesia administration [1: very unpleasant, 2: unpleasant, 3: satisfactory, 4: good, 5: very good], and complications (e.g. headache, high spinal, back pain, paresthesia, nausea and vomiting, failed spinal). All data was measured and recorded by an independent physician observer. Patient satisfaction data and documentation of complications were acquired via a written survey on the day after the cesarean delivery procedure.

Statistical analysis was performed using R. Categorical binary outcomes were assessed using the Chi-squared test [27]. Normally distributed data were compared using independent measures t-tests. Non-normally distributed data were evaluated using Wilcoxon-Mann-Whitney tests. Needle insertions and passes were analyzed using a Poisson regression model (using glm in R), which permitted calculation of rate ratios (RR) to estimate effect sizes for changes in number of insertions and passes (analogous to relative risk ratio) [28]. In all cases, a two-tailed p value<0.05 was taken to indicate statistical significance. Cohen’s d was computed to indicate effect sizes, with |d|=0.2 considered a small effect, |d|=0.5 a medium effect, and |d| ≥ 0.8 a large effect. At the time of protocol development, there were no existing studies on the use of Accuro in a clinical setting, nor were there studies evaluating the use of neuraxial ultrasound by resident physicians. As such, no comparable studies existed that could be used to establish the pre-study statistical power. Thus, we attempted to estimate the power by aggregating the results of existing studies that reported the outcome of successful dural puncture on the first needle insertion attempt [1,5,26]. Overall, we assumed an expected rate of first-attempt success of 40% in the palpation group and 73% in the ultrasound groups. Based on this assumption, 45 patients were required in each group to achieve a power of 0.9 and a type 1 error rate of less than 0.05.

## Results

**Figure 1:**
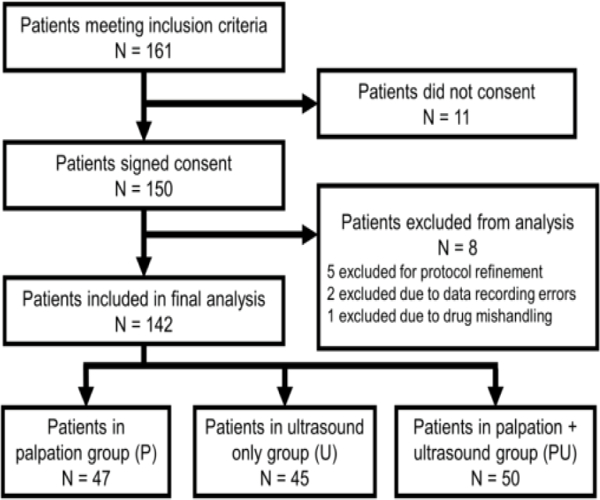
Flow diagram of subject management.

150 patients were recruited into the study (Figure 1). Eight patients were excluded for the following reasons: cases were performed prior to a protocol refinement that clarified when the attending physician should intervene (5 patients), data recording errors (2 patients), and drug spilled while injecting (1 patient). In total, 142 patients completed the study and no data was missing.

**Figure 2:**
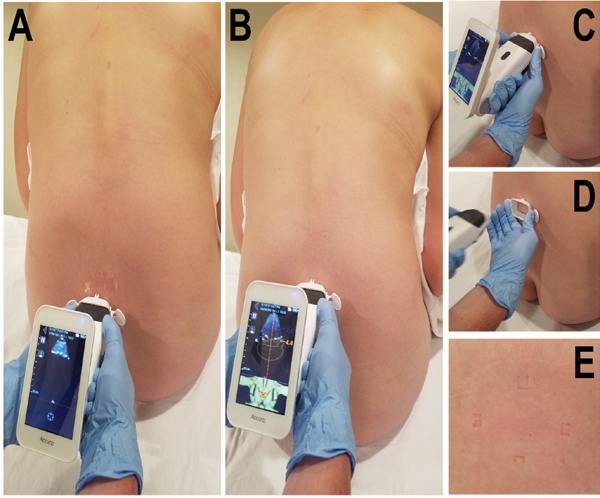
Sequential images of ultrasound imaging protocol used in this study. (A) First, the sacrum was identified as a bright horizontal structure when the device was placed just above the intragluteal cleft. (B) The system was advanced in the cephalad direction while maintaining the midline indicator in the center of the ultrasound viewport. (C) and (D) When the L4- L5 interspace was identified by the system, the skin was marked using the detachable Accuro Locator. (E) Four skin markings left by the Locator. The needle was placed at the center of the 4 indentations, which corresponded to the center of the ultrasound beam that intersected the L4-L5 interspace.

A sequence of images illustrating the use of Accuro is provided in Figure 2. Ultrasound images and SpineNav3D graphics for a patient of 79.6 kg/m^2^ BMI are shown in Video S1. Statistical analysis was performed across the P, U and PU groups and also for a combination of all cases in which ultrasound was used (AU: all ultrasound). The AU group was created after it was determined that study outcomes were similar between the U and PU groups. Accordingly, the majority of statistical analyses assessed differences between the P and AU groups.

Information related to patient characteristics and resident experience are summarized in Table 1. There were more obese subjects in the ultrasound groups (P *vs.* AU, p=0.027). Residents had performed a similar number of neuraxial procedures within each study group, and a post-hoc analysis indicated that outcomes were not significantly impacted by prior resident experience. Of the 95 cases in which Accuro was used, 41 were first-time uses (43%), 30 were second-time uses (32%), 18 were third-time uses (19%), 2 were fourth-time uses (2%), 2 were fifth-time uses (2%), and 1 was a sixth-time use (1%).

Results of primary and secondary outcomes across all subjects are presented in Table 2. First insertion success rates increased with the use of ultrasound (RR: 1.11 [0.85–1.47], p=0.431), although this was not statistically significant. Given the observed first insertion success rates of 59% in Group P and 66% in Group AU, the post-hoc statistical power of the study was 0.13, indicating that the study was underpowered to evaluate the primary endpoint.

Across all patients, use of ultrasound (AU) resulted in a 15% reduction in needle insertions (RR: 0.85 [0.71–1.00], p=0.052), a 26% decrease in needle passes (RR: 0.74 [0.53–1.05], p=0.110), and a 33% reduction in needle insertion time (Cohen’s d: −0.43 [(−0.79)-(−0.07)], p=0.070). It took longer to identify landmarks using Accuro (Cohen’s d: 1.24 [0.87–1.64], p<0.001), but the overall procedure time was not substantially different between groups. Two inadvertent dural punctures occurred in patients undergoing CSE in Group P. Overall, there were no significant differences in the rates of complications between the study groups.

A post-hoc analysis of spinal placement among obese patients (BMI ≥ 30 kg/m^2^) was performed (Table 3).

**Figure 3:**
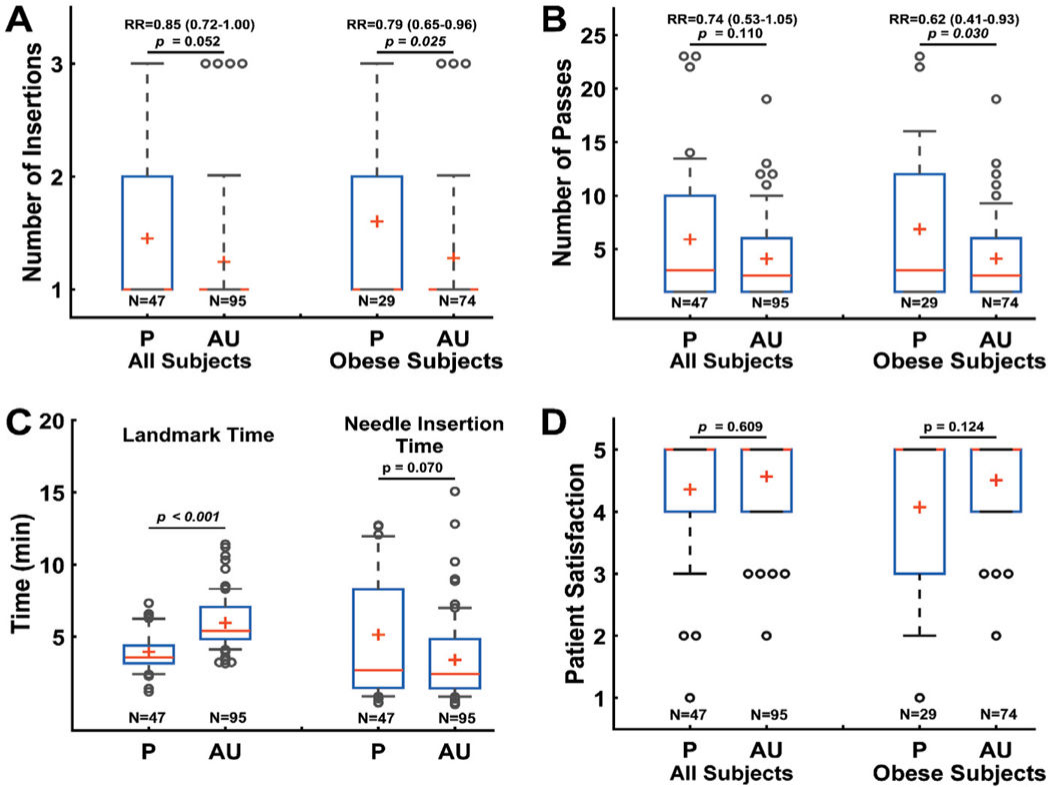
(A) Box plots of needle insertions required for spinal placement across all subjects and obese subjects. (B) Box plots of needle passes required for spinal placement across all subjects and obese subjects. (C) Box plots of time required for landmark identification and needle placement across all subjects. (D) Box plots of patient satisfaction scores across all subjects and obese subjects. In each box plot, the red ‘+’ is the arithmetic mean, the red horizontal line is the median, the extents of the blue box are the 25^th^ and 75^th^ percentile, the whiskers span between 10–90 % of the sample values, and the dots are outliers beyond the 10^th^ and 90^th^ percentiles. Rate ratios (RR) and their significance (p-values) are indicated for each comparison between the P (palpation only) group and AU (all ultrasound) groups. 95% confidence intervals for the value of RR are in parenthesis

First insertion success rates were 26% higher in Group AU versus Group P (RR: 1.26 [0.83–1.91], p=0.187). The use of ultrasound (AU) resulted in a 21% decrease in needle insertions (RR: 0.79 [0.65–0.96], p=0.025), a 38% decrease in needle passes (RR: 0.62 [0.41–0.93], p=0.030), a 58% reduction in difficult spinals (those requiring more than 10 passes, RR: 0.42 [0.21–0.85], p=0.011), a 57% reduction in needle insertion time (Cohen’s d=(−0.64)[−1.09–0.18]), and a 75% reduction in patients who reported patient satisfaction of less than 4 on a 5-point scale (RR: 0.25 [0.09–0.70], p=0.004). These results are depicted graphically in Figure 3.

**Figure 4:**
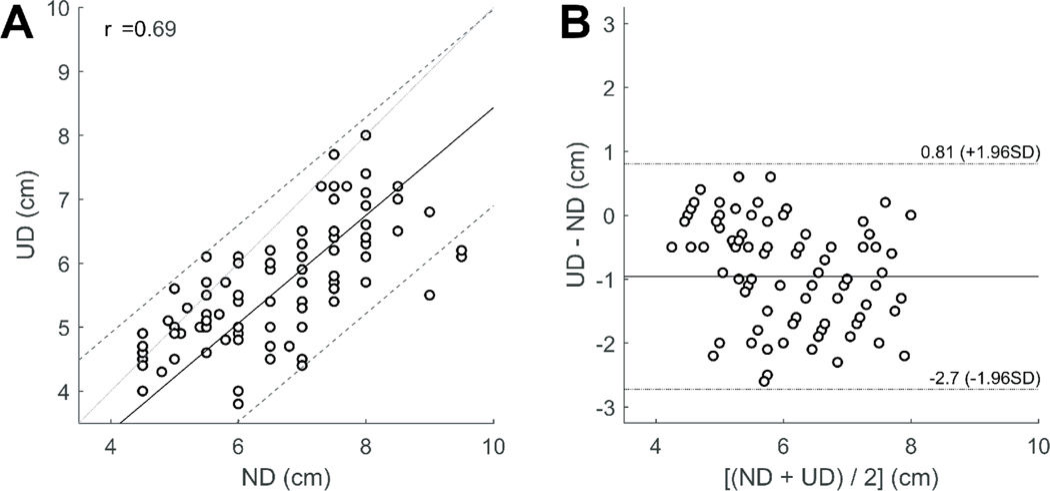
(A) Correlation plot of ultrasound depth versus needle depth at which CSF was visualized, Pearson correlation coefficient r=0.69 (95% CI:0.45–0.84, p<0.001). The thick line is the correlation and the dashed lines are the 95% confidence intervals. (B) Bland-Altman analysis of ultrasound depth *vs.* needle depth.

The correlation between the epidural depth automatically measured by Accuro (UD) and the needle depth at which CSF was visualized (ND) is shown in Figure 4A. The Pearson correlation coefficient r was 0.69 (95% CI: 0.45–0.84, p<0.001). A Bland-Altman analysis comparing the UD and ND is shown in Figure 4B. On average, the UD was 1.0 cm (95% CI: [0.81 - (−2.7)]) shallower than the ND, but it should be noted that Accuro measures the depth to the epidural space, not the depth to the deeper subarachnoid space [29].

## Discussion and Conclusion

Ultrasound has been widely investigated as a means for identifying neuraxial landmarks prior to performing lumbar neuraxial blockade procedures [6,7,13]. The benefits of preprocedural ultrasound imaging are particularly pronounced in the obese patient population, and several studies have demonstrated that both trainees [5,15,30] and experienced providers [1,14,24,31–33] achieve increased first-insertion success rates and make fewer skin punctures and needle passes when using ultrasound in patients with impalpable landmarks or high BMI. However, a common limitation of many of these studies is the reliance on experienced sonographers to perform the ultrasound examination, thereby limiting the relevance of the findings to routine clinical practice [1,5,24]. Moreover, blinding the care provider to the ultrasound examination results in incomplete use of the information that ultrasound provides, including the direction of needle angulation and detection of anatomical variations (e.g. scoliosis). Thus, it was the intent of this study to evaluate the feasibility of implementing neuraxial ultrasound in a cohort of resident physicians with minimal ultrasonography training. We hypothesized that the automated anatomical indicators provided to the practitioners by the dedicated neuraxial ultrasound system may compensate for their presumed training and experience gap.

First insertion success rates and most secondary endpoints were similar between the palpation and ultrasound groups. As in prior studies, the automated ultrasound system systematically underestimated the needle placement depth, with the largest discrepancies observed in obese patients [10,12]. The similar efficacy between the P, U, and PU groups suggests that the landmarks automatically identified by Accuro were functionally equivalent to those derived from palpation, thereby providing clinical validation of the accuracy of the automated landmark recognition technology. We had hypothesized that the combination of palpation and ultrasound in the PU group may have resulted in improved outcomes, but an improvement was not observed, again suggesting functional equivalence between the two landmark identification techniques in the context of this study.

As has been demonstrated in many prior studies [1,7,12–15,30–33], more pronounced differences in outcomes were observed when post-hoc sub-group analyses were performed on the outcomes for obese subjects. Reductions in the number of needle insertions, passes, and needle insertion time were observed, which may have contributed to the improved patient satisfaction scores reported by obese patients. In addition, the use of ultrasound resulted in a reduction in the number of cases requiring more than 10 needle passes, indicating that fewer cases required the use of exploratory needle passes [1]. While these findings must be considered merely suggestive of an overall trend, they are in broad agreement with a recent study conducted by Pan et al. [23] that demonstrated improved efficacy in spinal placement when using Accuro compared to palpation in obese parturients.

The results of this study also align with recent studies that have demonstrated the ability of novice practitioners to execute neuraxial ultrasonography following training regimens comprised of didactic teaching and supervised ultrasonography training [15,18]. Notably, while Arzola et al. [18] observed no benefit from the use of ultrasound in patients with easily palpable landmarks, Creaney et al. [15] demonstrated improved outcomes when novices used neuraxial ultrasonography in patients with impalpable spinous processes. In a departure from these prior studies, trainees in the present study received no supervised ultrasonography training and instead relied on automated interpretation of the ultrasound images to execute the neuraxial ultrasound procedure. Accuro’s automation was designed to promote a quick, task-based assessment of the neuraxial anatomy [21], and the ability of trainees to utilize this tool without expert supervision lends support to its use as a teaching aid. It is important for trainees to appreciate the three-dimensional anatomy of the lumbar spine when first learning to perform neuraxial techniques, and Accuro’s 2D and 3D anatomical renderings may expedite training without the need for direct expert supervision. However, additional study is required before establishing firm conclusions, especially as it pertains to effective training methods to enable optimal use of the technology.

The primary limitations of this study stem from it being among the first clinical trials to study the use of Accuro [10,23]. Little was known about the learning curve or clinical efficacy of Accuro prior to executing this study, which made it difficult to estimate the sample sizes required for a suitable a priori study power. Many results indicate a trend towards a benefit from the use of Accuro, but the lack of the required statistical power limits the strength of conclusions that may be drawn from this study. Additional limitations included the wide range in experience among the care providers in this study and the fact that resident physicians performed procedures within all study groups, which likely introduced additional variability and may have confounded direct evaluation of the impact of the Accuro technology on procedural and patient-related outcomes. Additionally, this study did not differentiate between CSE and single-shot spinals, which may have impacted results given the difference between the two procedures. Finally, lack of investigator and patient blinding may have introduced additional sources of bias.

In summary, this study suggests that resident trainees can implement neuraxial ultrasonography using Accuro without the need for didactic teaching or direct expert supervision. Use of neuraxial ultrasonography was likely the most beneficial in obese parturients, but no findings met the criteria for statistical significance. Further study is required to evaluate the efficacy of this technology when used by providers of different levels of expertise and in patients of variable body habitus and age.

## Supplementary Material

2

## Figures and Tables

**Table 1:** Demographic and clinical characteristics of the study subjects.

	P (N=47)	U (N=45)	PU (N=50)	AU (N=95)	p values P *vs.* AU
Patient Characteristics					
Age (year)	31.2 ± 5.2	30.0 ± 4.9	28.5 ± 5.5	29.1 ± 5.3	0.034^a^
BMI (kg/m^2^)	34.7 ± 7.7 [22.9–51.5]	36.6 ± 8.9 [27.6–79.6]	35.4 ± 9.3 [21.7–60.8]	36.0 ± 9.1 [21.7–79.6]	0.392^a^
Percent Obese	62%	89%	68%	78%	0.074^b^
Gestational Age (weeks)	38.4 ± 1.7	38.5 ± 1.5	38.4 ± 1.5	38.4 ± 1.5	0.980^a^
Percent receiving CSE	22%	20%	20%	20%	0.796^b^
First time receiving neuraxial anesthesia for birth	6%	22%	14%	18%	0.061^b^
Complications with prior neuraxial anesthesia	9%	6%	2%	4%	0.227^b^
Resident Characteristics					
Number of prior neuraxial procedures	51 [32–83]	51 [32–84]	54 [26–102.5]	57 [29–93.5]	0.943^c^
Complications					
Dural Puncture During CSE	4%	0%	0%	0%	0.142^b^
Back pain	4%	4%	2%	3%	0.756^b^
Nausea & Vomiting	9%	13%	10%	12%	0.592^b^

Data are reported as mean ± SD [range], percentile, or median [IQR], p-values derived from an undependent measures t-test,

b Chi-squared test,

c Wilcoxon Mann Whitney Test

**Table 2:** Outcomes reflecting efficacy of spinal anesthesia placement in all study subjects.

	P (N=47)	U (N=45)	PU (N=50)	AU (N=95)	p values	Effect Size
					P *vs.* AU	P *vs.* AU
**Technical Outcomes**						
1^st^ insertion success	59 (45–72) %	60 (44–74) %	74 (58–84) %	66 (56–76) %	0.431^a^	RR: 1.11 (0.85–1.47)
Number of insertions	1 [1,2]	1 [1,1]	1 [1,1]	1 [1,1]	0.052^b^	RR: 0.85 (0.71–1.00)
Number of passes	3 [1,8]	2 [1,6]	3 [1.75,6]	2.5 [1,6]	0.110^b^	RR: 0.74 (0.53–1.05)
Cases requiring>10 passes	28 (19–44) %	22 (11–38) %	8 (4–21) %	15 (9–24) %	0.070^a^	RR: 0.51 (0.26–0.99)
Landmarks time (min)	3.9 ± 1.4	6.0 ± 2.1	5.9 ± 1.5	6.0 ± 1.8	0.001^a^	d: 1.24 (0.87–1.64)
Needle insertion time (min)	5.0 ± 5.3	4.0 ± 3.4	3.0 ± 2.2	3.4 ± 2.8	0.070^c^	d: −0.44 (−0.80-(−0.08)
Total procedure time (min)	15.5 ± 6.3	17.6 ± 5.4	17.4 ± 7.0	17.5 ± 6.3	0.121^c^	d: 0.32 (−0.04–0.68)
**Patient Satisfaction**						
Score	5 [4–5]	5 [4–5]	5 [4–5]	5 [4–5]	0.609^d^	d: 0.27 (−0.11–0.63)
Score<4	19 (9–33) %	3 (1–15) %	12 (6–24) %	8 (4–16) %	0.083^a^	RR: 0.43 (0.17–1.12)

Data are reported as n % (95% CI), mean ± standard deviation, or median [IQR]. P-values derived from a Chi-squared test,

b Poisson regression model,

c Student’s t-test,

d Wilcoxon-Mann-Whitney test. Effect sizes are computed as Cohen’s d (standardized mean difference), relative risk for categorical data (RR), and rate ratios for Poisson regression model outputs (RR).

**Table 3:** Outcomes reflecting efficacy of spinal anesthesia placement in obese subjects.

	P (N=29)	U (N=40)	PU (N=34)	AU (N=74)	p values	Effect Size
					P *vs.* AU	P *vs.* AU
**Technical Outcomes**						
1^st^ insertion success	48 (31–64)%	60 (43–75)%	62 (44–78)%	61 (49–71)%	0.187^a^	RR: 1.26 (0.83–1.91)
Number of insertions	1 [1–2]	1 [1–1]	1 [1–1]	1 [1–1]	0.052^b^	RR: 0.79 (0.65–0.96)
Number of passes	4 [1–11.75]	2 [1–6]	3.5 [1–6]	2.5 [1–6]	0.030^b^	RR: 0.62 (0.41–0.93)
Cases requiring>10 passes	41 (26–60)%	22 (10–39)%	11 (4–27)%	17 (10–28)%	0.011^a^	RR: 0.42 (0.21–0.85)
Landmarks time (min)	3.9 ± 1.6	6.2 ± 2.0	5.7 ± 1.6	5.9 ± 1.8	<0.001^c^	d: 1.13 (0.67–1.61)
Needle insertion time (min)	6.4 ± 6.0	4.2 ± 3.8	3.4 ± 2.4	3.7 ± 3.1	0.047^c^	d: −0.64 (−1.09-(−0.18)
Total procedure time (min)	17.1 ± 6.9	18.1 ± 5.6	17.2 ± 8.1	17.7 ± 6.9	0.622^c^	d: 0.10 (−0.34–0.54)
**Patient Satisfaction**						
Score	4.5 [3–5]	5 [4–5]	5 [4–5]	5 [4–5]	0.124^d^	d: 0.57 (−0.08–0.99)
Score<4	31 (17–51)%	3 (1–17)%	12 (5–27)%	8 (4–18)%	0.004^a^	RR: 0.25 (0.09–0.70)

Data are reported as n% (95% CI), mean ± standard deviation, or median [IQR]. P-values derived from a Chi-squared test,

b Poisson regression model,

c Student’s t-test, d Wilcoxon-Mann-Whitney test. Effect sizes are computed as Cohen’s d (standardized mean difference), relative risk for categorical data (RR), and rate ratios for Poisson regression model outputs (RR).
