# Supplementary figures and images for: Feasibility of Spinal Anesthesia Placement Using Automated Interpretation of Lumbar Ultrasound Images: A Prospective Randomized Controlled Trial
Source: J Anesth Clin Res. Author manuscript; Available in PMC 2019 Jun 7. (PMC6555430; doi:10.4172/2155-6148.1000878)

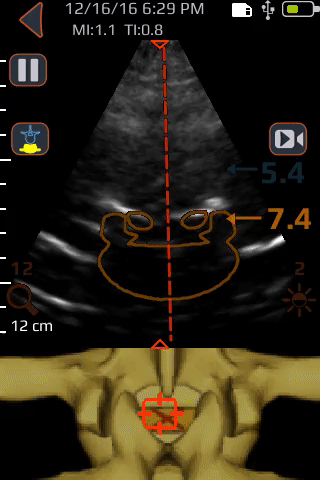

Supplement: 2 [file NIHMS1023603-supplement-2.gif]
